# Factors influencing health-related quality of life in patients with Type 1 diabetes

**DOI:** 10.1186/s12955-018-0848-4

**Published:** 2018-02-02

**Authors:** A. J. N. Raymakers, P. Gillespie, M. C. O’Hara, M. D. Griffin, S. F. Dinneen

**Affiliations:** 1 CÚRAM Centre for Research in Medical Devices, National University of Ireland Galway, Galway, Ireland; 2 Health Economics and Policy Analysis Centre, National University of Ireland Galway, Galway, Ireland; 3 Research and Development, Health Services Executive, Health and Wellbeing Division, Galway, Ireland; 4 School of Medicine, Clinical Science Institute, National University of Ireland Galway, Galway, Ireland; 5 Endocrinology and Diabetes Centre, Galway University Hospital, Galway, Ireland

## Abstract

**Aims:**

Generic, preference-based measures of health-related quality of life (HRQoL) are a common input to the economic evaluation of new health technologies. As such, it is important to explore what characteristics of patients with Type 1 diabetes might impact scores on such measures.

**Methods:**

This study utilizes baseline data from a cluster-randomized trial that recruited patients with Type 1 diabetes at six centers across Ireland. Health-related quality of life was assessed using the three-level EuroQol EQ-5D (EQ-5D) measure. Patients’ responses to individual dimensions of the EQ-5D were explored. To see which patient factors influenced EQ-5D scores, multivariate regression analysis was conducted with EQ-5D scores as the outcome variable.

**Results:**

Data was available for 437 Type 1 diabetes patients. The median age of these patients was 40 (IQR: 31-49) years and 53.8% were female. Overall, patients reported a high HRQoL based on EQ-5D scores (0.87 (SD: 0.19). Fifty-four percent of patients reported a perfect HRQoL. For those that reported problems, the most common dimension was the anxiety/depression dimension of the EQ-5D (29.6%). In the multivariate regression analysis, self-reported mental illness (− 0.22 (95% CI: -0.34, − 0.10)) and being unemployed (− 0.07 (95% CI: -0.13, − 0.02)) were negatively associated with EQ-5D scores (*p* < 0.05). The influence of self-reported mental illness was persistent in sensitivity analyses.

**Conclusions:**

The study results indicate that patients with Type 1 diabetes report a high HRQoL based on responses to the EQ-5D. However, there are a substantial number of Type 1 diabetes patients that report problems in the anxiety/depression dimension, which may provide avenues to improve patients’ HRQoL.

**Trial registration:**

Current Controlled Trials ISRCTN79759174.

**Electronic supplementary material:**

The online version of this article (10.1186/s12955-018-0848-4) contains supplementary material, which is available to authorized users.

## Introduction

The global incidence of Type 1 diabetes has been rising and is predicted to further increase in the foreseeable future [1]. Commensurate with this increase is the development and availability of new technologies for the monitoring and treatment of the disease [2]. Emerging technologies will need to demonstrate their relative value, compared to existing technologies, in order to be reimbursed by the relevant body. Economic evaluation is a method for making such comparisons, whereby the costs and benefits of new technologies, compared to the standard of care, are simultaneously considered [3]. Generic, preference-based measures of health-related quality of life (HRQoL) are often used to assess these benefits in economic evaluation and, thus, play a key role in the reimbursement of health interventions. As such, it is important to understand factors that might influence patients’ scores on these measures.

Existing literature for patients with Type 2 diabetes has found that obesity and the presence of diabetes-related complications were significant factors in determining patients EQ-5D scores [4]. For women with gestational diabetes, Danyliv et al. [5] found no statistically significant associations with patient characteristics and EQ-5D scores, aside from smoking status (specifically, being a previous smoker). In a study population of patients with Type 1 diabetes between 8 and 17 years of age, a significant association was estimated between poor glycemic control with both depression and HRQoL [6]. Poor glycemic control [7] and hyperglycemic symptoms [8] were also associated with reduced HRQoL, measured using the EQ-5D in adult patients, even after adjustment for diabetes-related complications [9]. Similar to results reported in patients with Type 2 diabetes, previous evidence suggests that diabetes-related complications are significantly and negatively related to EQ-5D index scores in adults with Type 1 diabetes [10].

Therefore, the objective of this study is to explore associations between those factors that influence HRQoL, as measured by the EQ-5D and EQ-VAS, in patients with Type 1 diabetes.

## Methods

This study used baseline data from the Irish Dose Adjustment for Normal Eating (DAFNE) cluster-randomized trial which compared structured curriculum-based group education follow-up with individual follow-up for patients with Type 1 diabetes. Full details are the trial are reported in the study protocol [11], clinical study [12], and cost-effectiveness analysis [13] Additional file 1.

### Health-related quality of life measurement

For this study, the outcomes of interest are the 3-level version of the EuroQol 5-dimension instrument (hereafter, the ‘EQ-5D’), and the secondary outcome, the EuroQol Visual Analogue Scale (EQ-VAS). The EQ-VAS accompanies the EQ-5D questionnaire and is a visual analogue scale for which respondents are directed to indicate their perceived HRQoL on that particular day (scale 0-100). The EQ-5D is a generic, preference-based measure of HRQoL consisting of five dimensions, each with three levels [14]. These five dimensions include: mobility, self-care, usual activities, pain/discomfort, and anxiety/depression. Each dimension has three effective levels: no problems, some problems, extreme problems. The EQ-5D responses were then used to compute a utility score which ranges from − 0.594 to 1. Where ‘1’ represents a state of ‘perfect’ health, ‘0’ is equal to death, and health states below ‘0’ are considered to be worse than death [15].

### Statistical analysis

We examined the distribution of EQ-5D index scores and EQ-VAS scores which revealed a skewed distribution for the EQ-5D scores, similar to what has been reported elsewhere [16]. EQ-VAS scores approximated a normal distribution. Univariate linear regression analyses were conducted using EQ-5D index scores and EQ-VAS scores as the outcome variable. Covariates were included in the multivariate model using a threshold of inclusion of *p* < 0.2 in these univariate models. Due to distributional concerns about EQ-5D scores, and concerns about the interpretability of results in a linear regression framework, we estimated the linear regression models using robust standard errors (also known as White-Huber standard errors) [17]. A sensitivity analysis was also performed, given these distributional concerns with EQ-5D scores, whereby a binary outcome variable was created for those reporting ‘any problems’ and those reporting ‘no problems’ in EQ-5D scores to explore whether the same associations existed in a logistic regression framework. Finally, we explored individual domain scores to determine whether covariates were significantly associated with specific domains of the EQ-5D, again by collapsing the responses into ‘any problems’ and ‘no problems’. All analyses were conducted using the statistical package ‘R’ (version 3.2.3).

## Results

This trial consisted of 437 patients with Type 1 diabetes from 6 centres across Ireland (including one site in Northern Ireland). The median age of these patients was 40 (IQR: 31-49) and 53.8% were female. Further details of the study patients are available in Table 1. Fifty-four percent of patients reported themselves as being in perfect health based on the EQ-5D index score. Only one patient reported a value of less than 0. The mean EQ-5D score and mean EQ-VAS score were 0.87 (SD: 0.19) and 71.5 (SD: 16.8), respectively.

**Table 1:** Characteristics of the study participants

Characteristic	Value
Age	40.8 (SD: 11.7)
Female	235 (53.8%)
Completed 3rd Level Education	178 (48.0%)
Single	142 (37.6%)
Employed	297 (78.4%)
Smoking History^a^	174 (45.6%)
Body Mass Index (BMI) kg/m^2^	26.0 (SD: 4.1)
Years Since Diagnosis	15.9 (SD: 10.8)
HbA_1c_ (%)	8.3% (SD: 1.4)
HbA_1c_ (mmol/mol)	67 (SD: 8)
Systolic BP mg/Hg	124.9 (SD: 18.9)
Diastolic BP mg/Hg	74.1 (SD: 10.9)
Complications^a^	93 (21.4%)
EQ-VAS^b^	71.5 (SD: 16.8)
EQ-5D^c^	0.87 (SD: 0.19)
Individual Dimensions of the EQ-5D	
Mobility	
No Problems	392 (89.7%)
Some Problems	35 (8.0%)
Extreme Problems	0 (0%)
Self-Care	
No Problems	416 (95.2%)
Some Problems	7 (1.6%)
Extreme Problems	0 (0%)
Usual Activities	
No Problems	354 (81.0%)
Some Problems	70 (16.3%)
Extreme Problems	3 (0.7%)
Pain/Discomfort	
No Problems	335 (78.3%)
Some Problems	81 (18.9%)
Extreme Problems	10 (2.3%)
Anxiety/Depression	
No Problems	295 (70.4%)
Some Problems	118 (28.2%)
Extreme Problems	6 (1.4%)

^a^ Patients reporting complications, including: neuropathy, foot ulcer, amputation, cardiovascular disease, proteinuria, retinopathy, blindness. ^b^ The EQ-VAS scale is 0 to 100. ^c^ The EQ-5D scale is − 0.594 to 1. *BMI* Body Mass Index, *HbA*_*1c*_ glycated hemoglobin, *HADS* Hospital Anxiety and Depression Score, *BP* Blood pressure, *EQ-5D* EuroQol 5-Dimension, *EQ-VAS* Visual Analogue Component of the EQ-5D instrument

### Individual EQ-5D dimensions

**Fig. 1:**
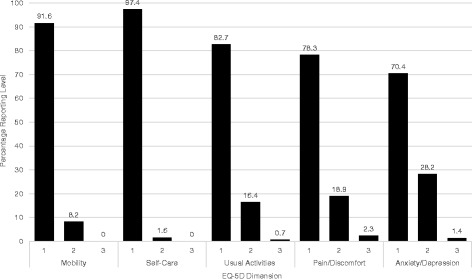
Percentage of patients reporting each level of EQ-5D dimension scores. Level 1 represents ‘No Problems’, level 2 ‘Some Problems’, and level 3 ‘Extreme Problems’

With regard to individual dimension scores, patients reported very few problems in the mobility and self-care dimensions (89.7% and 95.2%, respectively). In the dimensions reporting usual activities, pain/discomfort, and anxiety/depression, the number of patients reporting no problems was smaller (81.0%, 78.3%, and 70.4%, respectively). Figure 1 provides more detail on the scores for each dimension.

### Multivariate regression results

Multivariate regression results for EQ-VAS scores (scale 0-100) indicated that age, HbA_1c_, disease duration, and obesity (BMI ≥ 30 kg/m^2^) were significantly associated with patients’ HRQoL (all *p* < 0.05). Patients’ age had the smallest impact and obesity had the largest impact on EQ-VAS scores (0.21 ((95% CI: 0.04-0.39) and − 11.8 (95% CI: -17.2, − 6.3), respectively. Multivariate regression models were estimated to explore associations with EQ-5D index scores found that self-reported mental illness, and employment status, were significantly and negatively associated with EQ-5D index scores (*p* < 0.05 for both). Self-reported mental illness was associated with a reduction in EQ-5D index scores (scale 0-1) of approximately − 0.22 (95% CI: -0.34, − 0.10) and being unemployed was associated with a reduction of approximately − 0.07 (95% CI: -0.13, − 0.02) (Table 2). As a sensitivity analysis, EQ-5D respondents were categorized as having ‘no problems’ (EQ-5D equal to 1) or reporting any ‘problems’ (EQ-5D score less than 1) and a multivariate logistic regression analysis was conducted. The results of this analysis suggest that self-reported mental illness had the largest and statistically significant effect on reporting of problems with EQ-5D scores; that is, patients with self-reported mental illness were 8 times more likely to report problems in EQ-5D scores (*p* = 0.049). Mental illness was significantly associated with an increased likelihood of reporting problems in both the ‘Pain/Discomfort’ and ‘Anxiety/Depression’ domains (*p* < 0.05), and was not significantly associated with other domains. In addition, being unemployed was associated with an increased likelihood of reporting problems in the ‘Mobility’, ‘Self-Care’, and ‘Pain/Discomfort’ domains (*p* < 0.05).

**Table 2:** Regression analysis of baseline data with EQ-5D index scores (scale 0-1) as the outcome variable

	Estimate	95% LL	95% UL	*p* value
Age	−0.001	− 0.004	0.001	0.132
Gender (Male)	−0.011	− 0.052	0.029	0.568
Unemployed	−0.073	−0.130	− 0.016	0.039
Obese^a^	−0.046	−0.107	0.014	0.167
Medical Card	−0.040	−0.093	0.014	0.177
Marital Status (Single)	0.011	−0.032	0.055	0.580
Education^b^				
Complete 2nd level	0.010	−0.057	0.077	0.801
Complete 3rd level	0.014	−0.052	0.080	0.721
Complications^c^	−0.020	−0.073	0.032	0.407
Disease Duration	−0.002	−0.004	0.000	0.095
Mental Illness^d^	−0.219	−0.337	− 0.100	0.017
High Blood Pressure^d^	−0.009	−0.063	0.044	0.740
HbA_1c_ (%)	−0.018	−0.033	− 0.002	0.076
Heart Disease^d^	0.013	−0.103	0.129	0.886
Chest/Lung Disease^d^	0.051	−0.073	0.176	0.491

^a^ BMI ≥ 25 kg/m^2^. ^b^ Reference Category: did not complete second level education. ^c^ Patients reporting complications, including: neuropathy, foot ulcer, amputation, cardiovascular disease, proteinuria, retinopathy, blindness. ^d^ Categorical variable (Yes = 1, No = 0), self-reported. *LL* Lower limit of 95% confidence interval. *UL* Upper limit of 95% confidence interval

## Discussion

This study sought to explore associations between characteristics of Type 1 diabetes patients, with scores from a generic, preference-based measure of HRQoL, the EQ-5D. The importance of performing such exploratory analyses and understanding associations between patient characteristics and EQ-5D scores is two-fold. First, EQ-5D scores are often used in economic evaluations that inform reimbursement decisions for new health interventions. As such, it is important to understand the individual patient characteristics which form the basis for these scores. Second, individual EQ-5D dimension scores can be used to identify which dimensions have the greatest impact on patients’ overall HRQoL and these dimension scores might allow clinicians to understand where improvements could be made in patient care. The results of this study suggest many Type 1 diabetes patients do not report that their disease has an impact on their individual HRQoL, but, for a subset of Type 1 diabetes patients, mental illness (self-reported) has a statistically significant negative impact on HRQoL.

The results of the EQ-5D presented in this study might suggest that this generic measure of preference-based HRQoL may not be sufficiently sensitive to capture the impact of Type 1 diabetes on patients. However, it is worth noting that a five level version of the EuroQol instrument (EQ-5D-5L) has been developed to address the issue of sensitivity (as well as other issues) [18]. The descriptive system associated with the EQ-5D-5L is an obvious improvement to the EQ-5D-3L, but there remain issues about the tariffs used to estimate index scores, and how these calculations impact the results of cost-utility analyses [19]. Accordingly, there has been some reluctance from decision-making bodies to move toward recommending the EQ-5D-5L as a part of reimbursement submissions [20]. Similarly, the results reported in this study may have implications for the economic evaluation and reimbursement of new health technologies for individuals with diabetes. For example, if a new technology is put forward to be evaluated for reimbursement, unless it has the ability of improve patients’ mental health, and thus improve patients’ responses in the respective domains, it may not show any benefit for a given cost, and is potentially unlikely to be reimbursed. It also suggests that interventions targeting patients’ mental health are an avenue to improve their HRQoL. This result aligns with findings by Byrne et al. [21], whereby their analysis suggested that patients with higher levels of diabetes-related anxiety were most likely to benefit from support programs in terms of improved overall quality of life.

This study has several limitations. First, we used cross-sectional data from a previously completed trial to evaluate associations between HRQoL and potential influential factors. While it is certainly advantageous to use longitudinal data, we emphasize that this study was meant to be exploratory and form a basis for future research. Second, EQ-5D scores were asymmetrically distributed and, as such, pose concerns for standard regression techniques. However, we believe the techniques used to minimize the impact of these concerns are appropriate, particularly in the context of an exploratory study. Third, the presence of mental illness (and other comorbidities) was based on self-report. We acknowledge that a more robust ascertainment of clinical history would be an improvement, as would greater specificity as to the nature of mental illness.

## Conclusions

Type 1 diabetes patients enrolled in this study reported a high health-related quality of life, with over half of patients reporting ‘perfect’ health based on EQ-5D responses. However, in those that did report problems, the most common were reported in the ‘Anxiety/Depression’ dimension. Similarly, multivariate regression analysis suggested that self-reported mental illness had the most substantial impact on EQ-5D scores. The results of this study demonstrate the importance of exploring individual dimensions of EQ-5D scores and the findings may also imply that clinicians treating patients with Type 1 diabetes should be particularly mindful of their patients mental well-being.

## Additional files

Additional file 1: CONSORT Flow Diagram for the original cluster randomized controlled trial. (TIFF 6514 kb)

## Abbreviations

BMI: Body mass index
CI: Confidence interval
DAFNE: Dose Adjustment for Normal Eating
EQ-5D-3L: EuroQol 5-dimesion 3-level
HbA_1c_: Glycated hemoglobin
HRQoL: Health-related quality of life
IQR: Interquartile range
SD: Standard deviation
VAS: Visual analogue scale
