# Supplementary figures and images for: Factors influencing health-related quality of life in patients with Type 1 diabetes
Source: Health Qual Life Outcomes. 2018 Feb 2;16:27. doi: 10.1186/s12955-018-0848-4 (PMC5797407; doi:10.1186/s12955-018-0848-4)

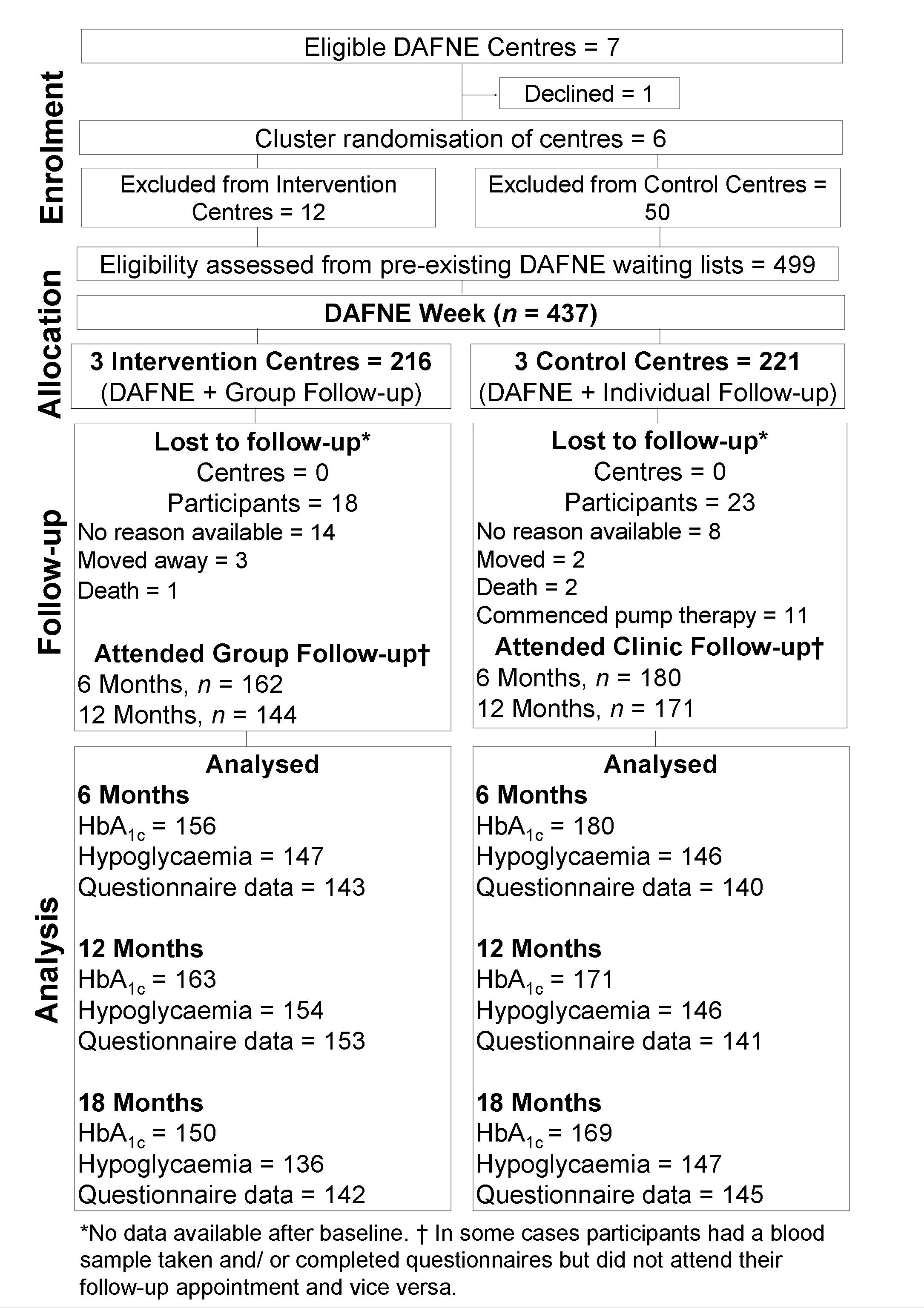

Supplement: Supplementary file 1 — CONSORT Flow Diagram for the original cluster randomized controlled trial. (TIFF 6514 kb) [file 12955_2018_848_MOESM1_ESM.tiff]
